# What is the empirical basis for converting banded ordinal data on numbers of sex partners among MSM into a continuous scale level variable? A secondary analysis of 13 surveys across 17 countries

**DOI:** 10.1186/s12874-021-01483-8

**Published:** 2022-03-06

**Authors:** Ana Mendez-Lopez, Ford Hickson, Klaus Jansen, Nathan Lachowsky, Fiona Burns, Cinta Folch, Annie Velter, Peter Weatherburn, Ulrich Marcus, Ursula von Rüden, Massimo Mirandola, Lorenzo Gios, Jamie Frankis, David J. Brennan, Axel J. Schmidt

**Affiliations:** 1 Department of Preventive Medicine, Public Health and Microbiology, School of Medicine, Autonomous University of Madrid, Madrid, Spain; 2 Sigma Research, Department of Public Health, Environments and Society, London School of Hygiene & Tropical Medicine, 15-17 Tavistock Place, London, WC1H 9SH UK; 3 Department for Infectious Disease Epidemiology, Robert Koch Institute, Berlin, Germany; 4 School of Public Health and Social Policy, University of Victoria, Victoria, Canada; 5 Community Based Research Centre, Vancouver, Canada; 6 Institute for Global Health, University College London, London, UK; 7 Centre d’Estudis Epidemiològics sobre les Infeccions de Transmissió Sexual i Sida de Catalunya (CEEISCAT), Agència de Salut Pública de Catalunya, Barcelona, Spain; CIBER Epidemiologia y Salud Pública (CIBERESP), Instituto de Salud Carlos III, Madrid, Spain; 8 Direction Prévention, Promotion de la santé, Agence nationale de santé publique, Saint Maurice, France; 9 Bundeszentrale für Gesundheitliche Aufklärung, Cologne, Germany; 10 Infectious Diseases Section, Department of Diagnostics and Public Health, University of Verona, Verona, Italy; 11 School of Health Sciences, University of Brighton, Brighton, UK; 12 School of Health and Life Sciences, Glasgow Caledonian University, Glasgow, UK; 13 Factor-Inwentash Faculty of Social Work, University of Toronto, Toronto, Canada

**Keywords:** Sexual behaviour, Men-who-have-sex-with-men, Survey research

## Abstract

**Background:**

To provide empirically based guidance for substituting partner number categories in large MSM surveys with mean numbers of sexual and condomless anal intercourse (CAI) partners in a secondary analysis of survey data.

**Methods:**

We collated data on numbers of sexual and CAI partners reported in a continuous scale (write-in number) in thirteen MSM surveys on sexual health and behaviour across 17 countries. Pooled descriptive statistics for the number of sexual and CAI partners during the last twelve (*N* = 55,180) and 6 months (*N* = 31,759) were calculated for two sets of categories commonly used in reporting numbers of sexual partners in sexual behaviour surveys.

**Results:**

The pooled mean number of partners in the previous 12 months for the total sample was 15.8 partners (SD = 36.6), while the median number of partners was 5 (IQR = 2–15). Means for number of partners in the previous 12 months for the first set of categories were: 16.4 for 11–20 partners (SD = 3.3); 27.8 for 21–30 (SD = 2.8); 38.6 for 31–40 (SD = 2.4); 49.6 for 41–50 (SD = 1.5); and 128.2 for ‘more than 50’ (SD = 98.1). Alternative upper cut-offs: 43.4 for ‘more than 10’ (SD = 57.7); 65.3 for ‘more than 20’ (SD = 70.3). Self-reported partner numbers for both time frames consistently exceeded 200 or 300. While there was substantial variation of overall means across surveys, the means for all chosen categories were very similar. Partner numbers above nine mainly clustered at multiples of tens, regardless of the selected time frame. The overall means for CAI partners were lower than those for sexual partners; however, such difference was completely absent from all categories beyond ten sexual and CAI partners.

**Conclusions:**

Clustering of reported partner numbers confirm common MSM sexual behaviour surveys’ questionnaire piloting feedback indicating that responses to numbers of sexual partners beyond 10 are best guesses rather than precise counts, but large partner numbers above typical upper cut-offs are common.

## Background

Survey designers and statisticians often have distinct needs when it comes to the choice of ordinal or continuous scale level variables for measuring sexual behaviours, for example, numbers of sexual or condomless anal intercourse (CAI) partners.

In sexual behaviour surveys with men who have sex with men (MSM), piloting survey questions on partner numbers has shown that men struggle with reporting precise partner numbers when they had ten or more partners in the past six or twelve months [1, 2]. Instead, respondents provide rounded estimates rather than precise counts when reporting numbers of partners beyond nine. For this reason, many surveys today use a mixture of continuous and ordinal scales, starting with a continuous scale format for partner numbers between 0 and 9 (or 10, or 19, or 20), and switching to categories thereafter.

For example, the European MSM Internet Survey (EMIS), the largest survey on sexual behaviour and sexual health among MSM worldwide, used the following answer format: 0, 1, 2, 3, 4, 5, 6, 7, 8, 9, 10, 11–20, 21–30, 31–40, 41–50, more than 50 for reporting sexual partner numbers [1]. However, statisticians often prefer calculating the attributable risk for each additional partner, for which a continuous scale variable is needed. The last category, be it ‘more than 10’, ‘more than 20’, or ‘more than 50’ is particularly difficult as the average number beyond this cut-off is unknown.

The aim of this study is to explore the clustering of reported numbers of sexual partners in studies with an open answer format, and to provide an empirical basis for substituting categories of partner numbers with their probable mean. This exercise allows us to explore the validity of alternative designs of survey questions and potential sources of bias in questionnaire design and respondents’ reporting in sexual behaviour surveys, providing methodological insights in sexual behaviour research.

## Methods

### Data sources

We collated data on number of sexual partners reported in a continuous scale in MSM surveys on sexual health and behaviour. We contacted epidemiologists and social researchers from the EMIS network [1–3] across Europe and Canada (typically at least one academic and/or governmental partner per country) to identify behavioural surveys among MSM conducted in their respective countries, and asked them which of the national surveys had used open write-in fields for the numbers of sexual partners. Thirteen national and multi-national surveys were identified as eligible, and data of the partner number write-in fields were obtained from these surveys and included in our analyses [4–15]. The surveys were undertaken between 1995 and 2019 across Europe and in Canada.

All surveys asked about the overall number of sexual partners, with four also asking specifically about the number of CAI partners. Eight surveys provided data for numbers of sexual partners over the previous 12 months and five for over the previous 6 months. Table 1 (header) lists all the surveys included, years and countries in which they were performed, type of data collected, and time frame (recall period).

**Table 1:** Pooled descriptive statistics for one set of bands for number of sexual partners: detailed estimates by survey, time frame and type of sexual partner

	**OVERALL**	**HIVHOM-1995–2006**	**GMA-2007**	**SEXHOM-2008**	**EPGL-2011**	**SexNow-2014/15**	**SMMASH-2016**	**London-GMSHS-2019**	**ERAS-2019**	**SMMASH-2016**	**London-GMSHS-2019**	**OVERALL**	**SIALON-2011**	**iCruise-2016**	**MSMSS-2018**	**SexNow-2019**	**ERAS-2019**	**SIALON-2011**	**MSMSS-2018**
**Countries**	AT, CA, DE, FR, ES, IE, UK	ES	AT, DE	ES	FR	CA	IE, UK-NIR, UK-SCT, UK-WLS	UK-ENG	FR	IE, UK-NIR, UK-SCT, UK-WLS	UK-ENG	BE, BG, CA, DE, FR, ES, IT, LT, PL, PT, RO, SE, SK, SI, UK	BE, BG, DE, ES, IT, LT, PL, PT, RO, SE, SK, SI, UK	CA	DE	CA	FR	BE, BG, DE, ES, IT, LT, PL, PT, RO, SE, SK, SI, UK	DE
**Partner number categories**	Sexual partners in the previous 12 months									CAI partners in the previous 12 months		Sexual partners in the previous 6 months						CAI partners in the previous 6 months	
Survey N	55,180	4,220	8,497	1,108	9,648	7,292	3,299	1,231	19,885	1,889	827	31,759	3,676	411	1,926	6,659	19,087	1,392	1,388
**Overall Mean**	**15.8**	33.7	12.8	23.0	18.8	12.2	13.9	14.4	13.1	5.3	8.2	**8.5**	10.2	11.3	10.2	10.4	7.1	4.8	7.3
N (11–20)	7,460	776	989	220	1,214	1,109	526	153	2,473	87	50	3,095	436	68	256	862	1,473	61	107
**Mean (11–20)**	**16.4**	16.7	16.5	16.6	16.9	15.9	16.2	17.2	16.2	15.8	17.2	**16.2**	16.8	14.6	16.5	16.0	16.1	16.5	16.6
N (21–30)	3,296	473	413	90	602	427	202	74	1,015	27	20	1,225	149	23	107	340	606	30	37
**Mean (21–30)**	**27.8**	27.8	27.9	27.3	28.1	27.2	27.5	27.9	27.9	27.4	28.4	**27.4**	27.8	26.0	28.7	27.5	27.1	27.0	28.6
N (31–40)	1,162	195	149	36	176	129	97	19	361	16	13	340	36	14	30	118	142	6	16
**Mean (31–40)**	**38.6**	38.8	38.4	38.5	38.8	37.9	38.2	38.4	38.8	37.3	38.3	**38.2**	38.9	35.6	39.0	38.1	38.2	37.2	38.9
N (41–50)	1,821	336	206	63	375	148	88	47	558	14	14	496	67	8	30	132	259	4	11
**Mean (41–50)**	**49.6**	49.5	49.7	49.7	49.7	49.1	49.3	49.9	49.6	47.9	49.7	**49.3**	49.8	46.6	49.8	48.7	49.5	48.8	50.0
N (>50)	2,912	653	338	83	625	208	107	57	841	20	17	536	90	11	48	130	257	11	23
*Sample Share*	*5.3%*	*15.5%*	*4.0%*	*7.5%*	*6.5%*	*2.9%*	*3.2%*	*4.6%*	*4.2%*	*1.1%*	*2.1%*	*1.7%*	*2.4%*	*2.7%*	*2.5%*	*2.0%*	*1.3%*	*0.8%*	*1.7%*
**Mean (>50)**	**128.2**	127.9	122.4	147.3	150.1	121.0	126.8	112.3	115.6	122.4	119.7	**107.3**	99.4	104.7	116.8	105.9	109.1	100.3	115.8
**Alternative upper cut-offs: **																			
N (>10)	16,651	2,433	2,095	492	2,992	2,021	1,020	350	5,248	164	114	5,692	778	124	471	1,582	2,737	112	194
*Sample Share*	*30.2%*	*57.7%*	*24.7%*	*44.4%*	*31.0%*	*27.7%*	*30.9%*	*28.4%*	*26.4%*	*8.7%*	*13.8%*	*17.9%*	*21.2%*	*30.2%*	*24.5%*	*23.8%*	*14.3%*	*8.0%*	*14.0%*
**Mean (>10)**	**43.4**	55.0	40.6	46.5	52.4	33.0	35.0	40.5	39.5	35.5	40.9	**31.4**	32.3	29.1	33.0	30.2	31.6	29.8	34.4
N (>20)	9,191	1,657	1,106	272	1,778	912	494	197	2'775	77	64	2,597	342	56	215	720	1,264	51	87
*Sample Share*	*16.7%*	*39.3%*	*13.0%*	*24.5%*	*18.4%*	*12.5%*	*15.0%*	*16.0%*	*14.0%*	*4.1%*	*7.7%*	*8.2%*	*9.3%*	*13.6%*	*11.2%*	*10.8%*	*6.6%*	*3.7%*	*6.3%*
**Mean (>20)**	**65.3**	73.0	62.2	70.6	76.6	53.7	55.0	58.6	60.3	57.8	59.3	**49.5**	52.1	46.8	52.7	47.3	49.6	45.7	56.3

*Note*: Respondents with missing data or reporting ‘zero’ partners were excluded from the overall N and thus from the overall mean and all sample share proportions. *EPGL* Enquête Presse Gays et Lesbiennes, *ERAS* Enquête Rapport au sexe, *CAI* condomless anal intercourse, *GMA* gay men and AIDS, *GMSHS* gay men sexual health survey, *HIVHOM* Conductas de riesgo en hombres que tienen relaciones sexuales con hombres reclutados por Internet, *MSMSS* MSM Screening Study, *SEXHOM* Monitorizacion bio-conductual en HSH (Catalunya), *SMMASH* Social Media, *MSM* Sexual and Holistic Health study

### Statistical analyses

Pooled descriptive statistics for the number of sexual partners over the previous 12 months were calculated, first, for the total number of survey respondents and, second, for two sets of commonly used categories for reporting number of sexual partners in sexual behaviour surveys. The first commonly used set of categories included the following bands: 11–20; 21–30; 31–40; 41–50; more than 50 partners (and more than 10; more than 20 as alternative upper cut-offs). The second set included 10–19; 20–29; 30–39; 40–49; 50 or more partners (and 10 or more; 20 or more as alternative upper cut-offs). These are two separate sets of categories with different cut-offs in their bands, thus providing different ranges (e.g., in the first set there is the category 10–19 sexual partners and in the second set there is the category 11–20 sexual partners).

Next, we calculated the mean number of sexual partners by time frame (over the previous 12 months vs. the previous 6 months), by survey (for each of the thirteen included surveys), and by type of sexual partner (any sexual partner vs. CAI partners) for one of the commonly reported set of categories to assess potential differences in the means by each of these aspects and to contrast the robustness of the first findings.

Respondents reporting ‘zero’ partners were excluded from the overall N and, thus, from the overall mean and all sample share proportions. Respondents with missing data on partner numbers were also excluded. The write-in answer ‘999’ (*N* = 29), which may have represented the researcher code ‘missing’, and the answers ‘1000’ and ‘2000’ (*N* = 11), which were extreme outliers, were excluded.

## Results

Across all surveys, the combined sample size of respondents reporting the number of sexual partners in the previous 12 and previous 6 months was 55,180 and 31,759, respectively.

**Fig. 1:**
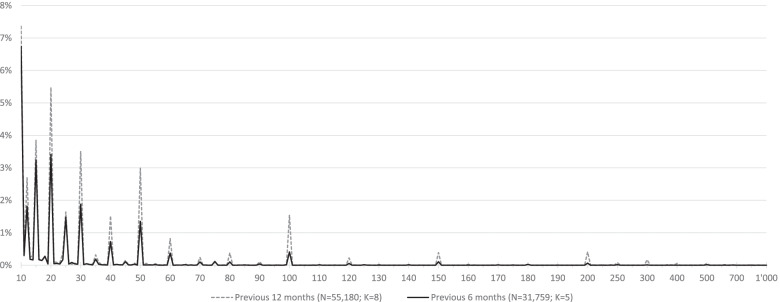
Distribution of overall male sexual partners among MSM (continuous scale variable). Data shown beyond nine partners only (N, number of respondents; K, number of surveys)

Figure 1 depicts the distribution of overall male sexual partners among MSM reporting more than nine partners. Across all surveys, the number of partners had an asymmetrical distribution skewed to the right. Self-reported partner numbers for both time frames consistently exceeded 200 or 300. Partner numbers beyond nine mainly clustered at 10, 12, 15, 20, 25, 30, 40, 50, 60, 80, 100, 120, 150, 200, and 300, regardless of the selected time frame.

Table 2 shows the total number and proportion of respondents reporting partners in each category, and pooled descriptive statistics (mean, standard deviation, median, and interquartile range) for each of the two alternative sets of bands. The pooled mean number of partners in the previous 12 months for the total sample was 15.8 partners (SD = 36.6), while the median number of partners was 5 (IQR = 2–15). Means for number of partners in the previous 12 months for the first set of categories were: **16.4** for 11–20 partners (SD = 3.3); **27.8** for 21–30 (SD = 2.8); **38.6** for 31–40 (SD = 2.4); **49.6** for 41–50 (SD = 1.5); and **128.2** for ‘more than 50’ (SD = 98.1). For the alternative upper cut-offs, mean partner numbers were **43.4** for ‘more than 10’ (SD = 57.7), and **65.3** for ‘more than 20’ (SD = 70.3).

**Table 2:** Pooled descriptive statistics for two sets of alternative bands for number of sexual partner categories: overall estimates for number of sexual partners for all the included MSM surveys

Categories for numbers of sexual partners: EMIS categories		Categories for numbers of sexual partners: alternative categories	
**Total**		**Total**	
N (%)	55,180 (100%)	N (%)	55,180 (100%)
Mean (SD)	15.8 (36.6)	Mean (SD)	15.8 (36.6)
Median (IQR)	5 (2–15)	Median	5 (2–15)
**11–20 partners**		**10–19 partners**	
N (%)	7460 (13.5%)	N (%)	8497 (15.4%)
Mean (SD)	16.4 (3.3)	Mean (SD)	12.1 (2.4)
Median (IQR)	15 (14–20)	Median	12 (10–15)
**21–30 partners**		**20–29 partners**	
N (%)	3296 (6.0%)	N (%)	4385 (8.0%)
Mean (SD)	27.8 (2.8)	Mean (SD)	21.5 (2.3)
Median (IQR)	30 (25–30)	Median	20 (20–24)
**31–40 partners**		**30–39 partners**	
N (%)	1162 (2.1%)	N (%)	2259 (4.1%)
Mean (SD)	38.6 (2.4)	Mean (SD)	30.7 (1.8)
Median (IQR)	40 (37–40)	Median	30 (30–30)
**41–50 partners**		**40–49 partners**	
N (%)	1821 (3.3%)	N (%)	1011 (1.8%)
Mean (SD)	49.6 (1.5)	Mean (SD)	40.9 (2.2)
Median (IQR)	50 (50–50)	Median (IQR)	40 (40–40)
**> 50 partners**		**≥ 50 partners**	
N (%)	2912 (5.3%)	N (%)	4561 (8.3%)
Mean (SD)	128.2 (98.1)	Mean (SD)	99.9 (86.9)
Median (IQR)	100 (75–150)	Median (IQR)	70 (50–100)
**Alternative upper cut-offs**			
**> 20 partners**		**≥ 20 partners**	
N (%)	9191 (16.7%)	N (%)	12,216 (22.1%)
Mean (SD)	65.3 (70.3)	Mean (SD)	54.1 (64.1)
Median (IQR)	48 (30–70)	Median (IQR)	30 (21–50)
**> 10 partners**		**≥ 10 partners**	
N (%)	16,651 (30.2%)	N (%)	20,713 (37.5%)
Mean (SD)	43.4 (57.7)	Mean (SD)	36.8 (53.4)
Median (IQR)	25 (16–50)	Median (IQR)	20 (12–40)

Notes: *SD* Standard Deviation, *IQR* Inter-quartile Range

Means for the second set of commonly reported categories were: **12.1** for 10–19 partners (SD = 2.4); **21.4** for 20–29 (SD = 2.3); **30.7** for 30–39 (SD = 1.8); **40.9** for 40–49 (SD = 2.2); and **99.9** for ‘50 or more’ (SD = 86.9). For the alternative upper cut-offs, mean partner numbers were **36.8** for ‘10 or more’ (SD = 53.4); **54.1** for ‘20 or more’ (SD = 64.1).

Mean number of sexual partners for the first set of categories (those whose bands commenced with a multiple of ten, e.g. 30–39) consistently resulted in a lower mean compared to the alternative band (those containing a multiple of ten in the upper end of the range, e.g. 31–40). According to the right skewed distribution (Fig. 1), all reported medians are consistently lower than the reported means (Table 2).

Table 1 shows, for each survey considered, the numbers and proportions of respondents reporting partners in each category, and the mean number of partners reported within each category, for both the previous 12 months and previous 6 months, and for both number of sexual partners and number of CAI partners. These data are provided only for one of the commonly used set of categories.

While there was substantial variation across all surveys with respect to the overall mean numbers, the means for all categories were very similar.

Across the two time frames, the overall mean number of sexual partners in the previous 6 months was 54% of the overall mean partner number in the previous 12 months. When moving towards higher partner number categories, this proportion increased steadily to 72% (‘more than 10’), 76% (‘more than 20’), and 84% (‘more than 50’).

Irrespective of the time frame, in all surveys providing estimates for the number of sexual and CAI partners, the overall means for CAI partners were lower than those for sexual partners. Such difference was absent from all categories beyond ten sexual and CAI partners.

## Discussion

Using data from thirteen national and multi-national sexual behaviour surveys among MSM, we looked at the distribution of partner numbers in the previous 12 and 6 months and calculated means for two commonly used sets of partner number categories.

The means and medians for the two sets of commonly reported categories show very different results because of clustering of responses in the tens, which pulled down, or up, the mean and medians toward the tens, whether it was included in the upper or lower limit of the band range. Clustering of reported partner numbers confirm MSM sexual behaviour surveys’ questionnaire piloting feedback, such as that from the EMIS [1, 2], that partner numbers beyond nine—and irrespective of the chosen time frame—were best guesses rather than precise counts, with ‘twelve’, ‘60’, and ‘120’ possibly reflecting ‘about one per month’, ‘about five per month’, and ‘about ten per month’, respectively, in a 12 months retrospective period.

The decreasing difference between means of partner numbers in higher categories across the two time frames may be due to the so-called telescoping effect, a cognitive effect in survey research, whereby there is a temporal displacement of events [16]. In this case events may be perceived as having happened some time nearer or further from the time of interview. This consistently occurred across all included surveys, and this effect may have slightly inflated the reported number of partners in the higher categories of surveys querying about the previous 6 months.

Above nine partners, the mean numbers of sexual and CAI partners were rather similar, suggesting that non-condom use is intermittent during anal sex with a smaller number of partners, but may become more common when dealing with larger numbers of sexual (intercourse) partners.

We found that for the higher categories in both sets of bands, i.e. ‘more than 50’ / ‘50 or more’, the associated interquartile ranges were wide and the standard deviations were high in relation to the means (Table 2). Given the large dispersion of the distribution in the highest categories of both sets of bands, adding an additional category in the upper range of the sets (for example, a category measuring 51–100 / 50–99 sexual partners and an additional capturing ‘more than 100’ / ‘100 or more’ sexual partners), may provide greater precision in measurement in surveys collecting data with categorical variables. Greater accuracy in the estimation of the number of sexual partners in the MSM population may contribute to overcoming difficulties in the prevention and control of the HIV/STI epidemics and their risk assessment.

One limitation of this analysis is that our findings may not transfer to MSM behaviour in other countries outside the regions of the countries from the surveys, especially those countries where homosexuality is highly stigmatised. Another limitation is that our selection of MSM surveys was not strictly systematic, however, given the composition of the research network, we consider it unlikely that we missed large European or Canadian surveys that no-one in the network was aware of. Additionally, the time lapse between the earliest and latest surveys is of almost 25 years, a time period throughout which MSM sexual behaviour may have changed due to, for instance, developments in HIV/STI interventions or cultural changes related to reduced stigmatization. Nonetheless, we do not expect the potential omission of a survey or the time lapse between the included surveys to have substantially impacted on the overall results. The large number of surveys included makes our study a comprehensive review and summary of the sexual behaviour in terms of number of sexual partners of MSM in Canada and Europe.

## Conclusions

The variations of the calculated means across surveys conducted in different countries, study populations and years were low. Therefore, we believe that the results can serve to foster methodologically robust substitution of partner number categories with probable mean numbers of sexual and CAI partners in MSM-oriented surveys.

## Data Availability

The datasets used and/or analysed during the current study are available from the corresponding author on reasonable request.

## Abbreviations

CAI: Condomless anal intercourse
EMIS: European MSM Internet Survey
MSM: Men-who-have-sex-with-men

## References

[1] Weatherburn P, Hickson F, Reid DS, Marcus U, Schmidt AJ. European men-who-have-sex-with-men internet survey (EMIS-2017): design and methods. Sex Res Soc Policy. 2020;17:543–57. 10.1007/s13178-019-00413-0.

[2] Weatherburn P, Schmidt AJ, Hickson F, Reid D, Berg RC, Hospers HJ (2013). The European men-who-have-sex-with-men internet survey (EMIS): design and methods. Sex Res Soc Policy.

[3] EMIS network (2017). European Men-who-have-sex-with-men Internet Suvey 2017 website.

[4] Folch C, Muñoz R, Zaragoza K, Casabona J (2009). Sexual risk behaviour and its determinants among men who have sex with men in Catalonia, Spain. Eurosurveillance..

[5] Méthy N, Velter A, Semaille C, Bajos N (2015). Sexual behaviours of homosexual and bisexual men in France: a generational approach. PLoS One.

[6] Gios L, Mirandola M, Toskin I, Marcus U, Dudareva-Vizule S, Sherriff N (2016). Bio-behavioural HIV and STI surveillance among men who have sex with men in Europe: the Sialon II protocols. BMC Public Health.

[7] Frankis J, Flowers P, McDaid L, Bourne A (2018). Low levels of chemsex among men who have sex with men, but high levels of risk among men who engage in chemsex: analysis of a cross-sectional online survey across four countries. Sex Health.

[8] Logan L, Fakoya I, Howarth A, Murphy G, Johnson AM, Rodger AJ (2019). Combination prevention and HIV: a cross-sectional community survey of gay and bisexual men in London, October to December 2016. Euro Surveill.

[9] Card KG, Fournier AB, Sorge JT, Morgan J, Grace D, Ham D (2020). Substance use patterns and awareness of biomedical HIV prevention strategies among sexual and gender minority men in Canada. AIDS Care.

[10] Jansen K, Steffen G, Potthoff A, Schuppe A-K, Beer D, Jessen H (2020). STI in times of PrEP: high prevalence of chlamydia, gonorrhea, and mycoplasma at different anatomic sites in men who have sex with men in Germany. BMC Infect Dis.

[11] Duchesne L, Lydié N, Velter A (2020). Increase in the overall level of protected anal sex in men who have sex with men in France: results from the repeated cross-sectional survey rapport au Sexe, France, 2017-2019. AIDS Care.

[12] Brennan DJ, Kesler M, Lachowsky NJ, Davies A, Georgievski G, Adam BD, et al. Sociodemographic and Psychological Predictors of Seeking Health Information Online among GB2M in Ontario: Findings from the #iCruise Project, International Journal of Sexual Health. 2021. 10.1080/19317611.2021.2000087. 10.1080/19317611.2021.2000087 PMC10903557 38596527

[13] Bochow M, Schmidt AJ, Grote S (2010). Schwule Männer und HIV/AIDS: Lebensstile, Szene, Sex 2007. Ein Befragung im Auftrag der Bundeszentrale für gesundheitliche Aufklärung, Köln.

[14] Centre d'Estudis Epidemiològics sobre la Sida de Catalunya (CEESCAT) (2011). Informe epidemiològic biennal CEEISCAT. Sistema integrat de vigilància epidemiològica de la SIDA/VIH/ITS a Catalunya. SIVES 2010.

[15] Ferlatte O, Salway T, Trussler T, Oliffe JL, Gilbert M (2018). Combining intersectionality and syndemic theory to advance understandings of health inequities among Canadian gay, bisexual and other men who have sex with men. Crit Public Health.

[16] Kupek E (2002). Bias and heteroscedastic memory error in self-reported health behavior: an investigation using covariance structure analysis. BMC Med Res Methodol.

